# Expanding the Neuron's Calcium Signaling Repertoire: Intracellular Calcium Release via Voltage-Induced PLC and IP3R Activation 

**DOI:** 10.1371/journal.pbio.0050066

**Published:** 2007-03-06

**Authors:** Stefanie Ryglewski, Hans J Pflueger, Carsten Duch

**Affiliations:** 1 Institute of Biology/Neurobiology, Free University of Berlin, Berlin, Germany; 2 School of Life Sciences, Arizona State University, Tempe, Arizona, United States of America; University of California San Diego, United States of America

## Abstract

Neuronal calcium acts as a charge carrier during information processing and as a ubiquitous intracellular messenger. Calcium signals are fundamental to numerous aspects of neuronal development and plasticity. Specific and independent regulation of these vital cellular processes is achieved by a rich bouquet of different calcium signaling mechanisms within the neuron, which either can operate independently or may act in concert. This study demonstrates the existence of a novel calcium signaling mechanism by simultaneous patch clamping and calcium imaging from acutely isolated central neurons. These neurons possess a membrane voltage sensor that, independent of calcium influx, causes G-protein activation, which subsequently leads to calcium release from intracellular stores via phospholipase C and inositol 1,4,5-trisphosphate receptor activation. This allows neurons to monitor activity by intracellular calcium release without relying on calcium as the input signal and opens up new insights into intracellular signaling, developmental regulation, and information processing in neuronal compartments lacking calcium channels.

## Author Summary

In neurons, calcium ions play a dual role as charge carriers and intracellular messengers, thereby linking brain activity to cellular changes. Alterations in the electrical potential across the cell's outer membrane (as happens, for example, when a neuron fires an action potential), can induce an influx of calcium ions through voltage-dependent membrane channels, which in turn regulate multiple cellular processes, such as gene transciption, cytoskeletal rearrangements, or even cell death. Stores of calcium ions also exist within neurons, which release their contents in response to multiple intracellular signals, including calcium itself. Here, we demonstrate that the neuronal cell membrane also possesses a voltage sensor that activates an intracellular calcium-release mechanism. This sensor enables neurons to recruit intracellular calcium signaling pathways in response to electrical activity without relying on calcium channels in their membrane. As large parts of a neuron's membrane may not contain calcium channels, this novel mechanism adds previously unanticipated calcium signaling possibilities to the neuron's intracellular communication machinery.

## Abstract 3

Ryglewski et al. identify a novel dependency of intracellular calcium release in neurons that requires G-protein activation upon plasma membrane depolarization.

## Introduction

Neuronal calcium plays a dual role as a charge carrier and as an intracellular messenger. Calcium signals regulate various developmental processes, such as migration in the central nervous system (CNS) [1], growth-cone behavior [2], dendritic development [3,4], and synaptogenesis [5], but calcium is also involved in apoptosis [6], and it regulates neurotransmitter release and membrane excitability [7]. How can one ubiquitous intracellular messenger regulate so many different vital processes in parallel, but independently? The answer lies in the versatility of the calcium signaling mechanisms in terms of amplitude and spatiotemporal patterning within a neuron [8], and the present study adds a novel mechanism to the bouquet of neuronal calcium signals.

The neuronal plasma membrane contains numerous voltage-operated (VOCs), receptor-operated (ROCs) and store-operated (SOCs) calcium channels carrying out different functions in different parts of the cell. For example, in vertebrate neurons, N- and P/Q-type VOCs trigger vesicle fusion at synaptic terminals, whereas L-type VOCs are located proximally to provide calcium signals regulating gene transcription. Structural plasticity of dendrites and filopodia is mediated via calcium influx through VOCs and ROCs and also by local release from internal stores [5,9]. Internal calcium stores are held within the membrane system of the endoplasmic reticulum (ER). Release from the ER is mediated by the inositol 1,4,5-trisphosphate receptor (IP3R) and the ryanodine receptor (RYR) families, both of which depend on the concentration of free intracellular calcium ([Ca^2+^]_i_ [10]). The patterns of calcium signals depend on the distributions of the calcium entry mechanisms and calcium-binding proteins within a specific neuronal compartment [8]. Calcium buffers, such as parvalbumin and calbindin, can locally confine calcium signals even to individual synapses [11]. Calcium-sensing proteins translate elevation or even temporal patterns in [Ca^2+^]_i_ into diverse cellular processes [7,12].

In summary, different spatiotemporal distributions of calcium signals, buffers, and sensors in various neuronal compartments allow distinct calcium signals to be assigned separately to specific cellular processes. Conversely, multiple calcium signals can act as coincidence detectors [13]. Nevertheless, to date, no mechanism is known by which a neuron can monitor its own activity by ER calcium release in the absence of both metabotropic receptor activation and calcium influx from the extracellular space. Consequently, influx and store activation are thought to be irrevocably linked, such that they neither operate independently from each other, nor work in concert for coincidence detection. In the present study, we use well-characterized and identified insect central neurons, the dorsal unpaired median (DUM) neurons, as a model to analyze a novel calcium-release mechanism in neurons. DUM neurons are among the best-studied insect neurons that can be individually identified in culture [14,15]. Therefore, they are well suited for pharmacological analysis of cellular signaling mechanisms. We demonstrate the existence of a novel voltage-dependent, but not calcium-dependent, mechanism of IP3 production, by simultaneous patch clamping and calcium imaging. This mechanism requires voltage-dependent, but not receptor-dependent, G-protein activation, which in turn, leads to phopholipase C activation, IP3 production, and calcium release from internal stores.

## Results

### Depolarization-Induced Intracellular Calcium Signals Occur in the Absence of Calcium Influx

**Figure 1:**
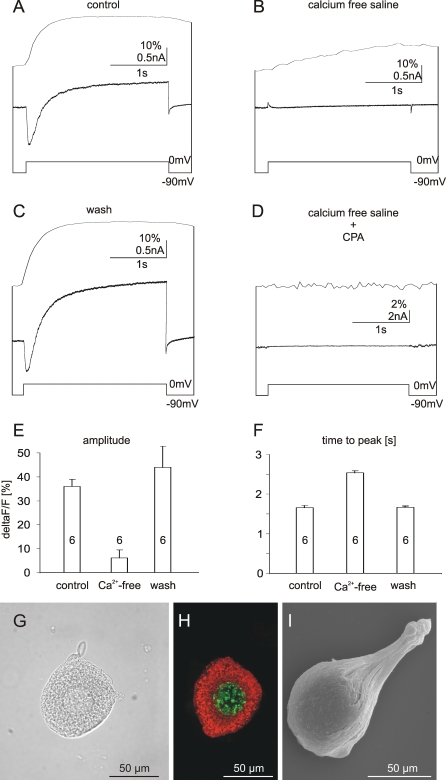
Voltage-Induced Intracellular Calcium Signals Occur in the Absence of Calcium Inward Current (A–C) Simultaneous patch clamp recording (lower traces) and calcium imaging (upper traces) from a representative, acutely isolated DUM neuron under different conditions. (A) In TEA- and TTX-containing saline, a voltage step from −90 mV holding to 0 mV test potential causes a calcium inward current followed by a calcium-activated potassium outward current. This is accompanied by a large elevation in internal calcium. (B) No membrane currents are observed in the same neuron after 3 min in calcium-free saline, but a slow elevation in intracellular calcium occurs in response to a similar voltage step as in (A). (C) Switching back to calcium-containing extracellular saline restores membrane currents and intracellular calcium signals as observed in (A). (D) Following depletion of intracellular calcium stores by extracellular application of CPA, no calcium elevation is recorded in zero-calcium saline in response to the step depolarization. (E and F) Average amplitudes (E) and average times to peak (F) of the calcium signals are shown for six neurons (mean and standard deviation) that were recorded continuously in calcium-containing control saline (left bars), zero-calcium saline (middle bars), and calcium-containing saline (right bars). (G) Photograph of a representative neuron just prior to recording demonstrates that isolated neurons are not in contact with each other or with neighboring glia cells. (H) This lack of contact is further supported by immunocytochemical labeling of the same neuron after the physiological experiments. Anti HRP (red) is used as a general neuronal marker, and a DNA stain (propidium iodide and RNAse [green]; see Materials and Methods) is used to depict nuclei. No nuclei of glia cells or neurons are detected in proximity to the recorded neuron. (I) SEM picture of a recorded soma with its primary neurite shows no contact with other neighboring cells.

Simultaneous patch clamp and optical recordings of freshly isolated DUM neurons were conducted under pharmacological block of voltage-activated potassium and sodium currents. Consequently, voltage steps of 2.5-s duration from a holding potential of −90 mV to 0 mV induced a large transient voltage-activated calcium inward current followed by a calcium-activated potassium current (Figure 1A, middle trace), both of which have previously been described in detail [15]. Simultaneous optical recordings of the changes in fluorescence of the calcium indicator Oregon Green Bapta (percent change as ΔF/F) revealed a long-lasting increase in ([Ca^2+^]_i_ which outlasted the calcium inward current; Figure 1A, top trace). The kinetics and the amplitude of the calcium signal suggest calcium release from intracellular stores in addition to calcium influx through VOCs, as is typical for most neurons. Both the calcium current and the calcium-activated potassium current were completely abolished after 3 min in zero-calcium (calcium replaced by magnesium, 1 μM EGTA) saline (Figure 1B, middle trace). Strikingly, the neurons still showed an increase in calcium indicator fluorescence as a response to the same voltage step from −90 mV holding to 0 mV (Figure 1B, top trace) in the absence of any calcium inward current. This voltage-induced calcium signal also remained after bath application of calcium-free saline with 500 μM cadmium to block all voltage-activated calcium channels (unpublished data). The time course was considerably slower and the amplitude was significantly smaller as compared to the intracellular calcium signal in the presence of calcium inward current (compare Figure 1A and 1B, top traces). Washing with calcium-containing saline led to a complete recovery of the initial calcium signal within 3 min (compare Figure 1A and 1C). After depletion of intracellular calcium stores by bath application of cyclopiazonic acid (CPA; 20μM), which has been demonstrated to block the SERCA pump in insect neurons [16], no increase in fluorescence was evoked by a depolarizing voltage step in calcium-free saline (Figure 1D). These data demonstrate calcium release from intracellular stores as a response to membrane depolarization without calcium influx from the extracellular space. Quantitatively, in the presence of extracellular calcium, a voltage step from −90 mV to 0 mV induced an increase in fluorescence of 36 ± 3.4% (mean and standard deviation) that peaked after 1.67 ± 0.05 s. Without calcium influx through VOCs, the neurons responded with fluorescence increases of 6.3 ± 3.0% and the time to peak was considerably longer (2.53 ± 0.05 s; Figure 1E). To make sure that the calcium signals observed were caused by membrane depolarizations of the recorded neurons, and not by interactions between neighboring cells, recordings were performed from completely isolated neurons not in contact with any other cell in the dish. Living neurons were imaged in bright field mode just prior to the physiological tests to demonstrate that no cell–cell contacts existed (Figure 1G). This was further supported by immunocytochemical labeling of the same neurons after the physiological experiments (Figure 1H). No nuclei of glia cells or neurons were detected with confocal microscopy in proximity to the recorded neuron, nor did a general neuronal marker detect any small cells nearby (resolution is 200 × 200 × 300 nm). And finally, scanning electron micrograph (SEM) pictures clearly demonstrated that neither the somata nor the primary neurites of the recorded neurons were in contact with any other neighboring cell. Therefore, the calcium signals occurring in the absence of extracellular calcium were due to membrane depolarizations of the recorded neurons and not to the release of signaling substances from nearby cells.

**Figure 2:**
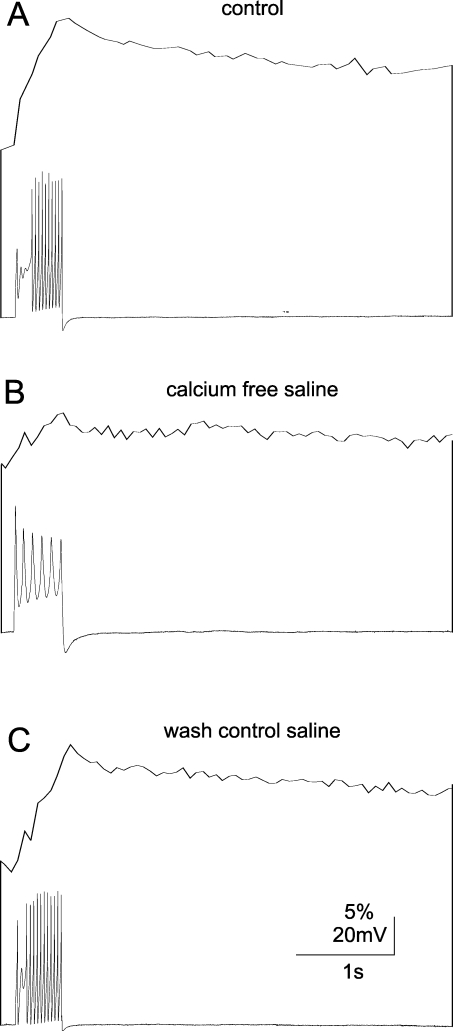
Bursts of Action Potentials Induce Intracellular Calcium Elevations in the Absence of Calcium Influx (A) In normal extracellular saline, a burst of action potentials (lower trace) induced by current injection of 0.5 nA is accompanied by a large elevation in intracellular calcium (upper trace). (B) Action potential shape and frequency are altered after 3 min in zero-calcium extracellular saline, but bursts are still accompanied by an elevation in intracellular calcium, although the calcium signal is slower and of lower amplitude as compared to (A). (C) Switching back to normal saline for 3 min restores signals as observed in (A).

To test whether spiking patterns of DUM neurons, as occurring during normal behavior [17,18], were sufficient to produce [Ca^2+^]_i_ elevations in the absence of calcium influx, a similar protocol as depicted in Figure 1 was repeated under current clamp conditions (Figure 2). First, in calcium-containing saline, bursts of action potentials were induced by current injection of 500-ms duration just above firing threshold. Individual bursts caused an increase in fluorescence of up to 15% (Figure2A, top trace). Second, after 3 min in calcium-free saline, action potential amplitude was reduced due to the lack of calcium influx, and action potential duration was increased due to the lack of calcium-activated potassium current. Consequently, spike frequency within the burst was also reduced (Figure 2B, bottom trace). However, under calcium-free conditions, bursts of only six action potentials were sufficient to produce intracellular calcium signals of up to 5% amplitude, which followed a slower time course as compared to control conditions (compare Figure 2A and 2B). Washing in normal calcium-containing saline led to a complete recovery of action potential shape and frequency and of the initial calcium signal (Figure 2C). This showed that the spiking activity that occurs during normal behavior without blocking sodium or potassium channels induced calcium release from internal stores without calcium influx through VOCs.

### Voltage-Induced Calcium Signal Depends on IP3R and PLC Activation, but Not on RYRs

**Figure 3:**
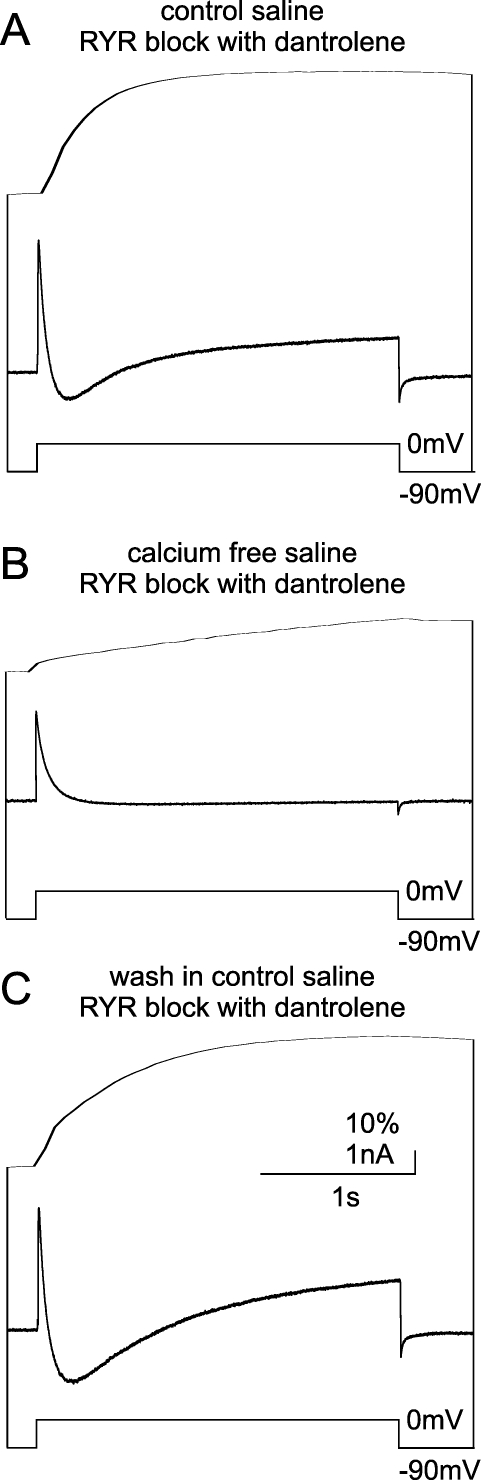
Voltage-Induced Intracellular Calcium Signals That Occur in the Absence of Extracellular Calcium Do Not Depend on RYRs (A) In dantrolene-loaded, acutely isolated DUM neurons in TEA- and TTX-containing saline, a voltage step from −90 mV holding to 0 mV test potential causes a calcium inward and a calcium-activated potassium outward current accompanied by an intracellular calcium signal. (B) After 3 min in calcium-free saline, a slow elevation in intracellular calcium occurs in response to the same voltage step as in (A), although RYRs are blocked by intracellular application of dantrolene (100 nM). (C) Switching back to calcium-containing extracellular saline restores membrane currents and intracellular calcium signals as observed in (A).

In principle, in neurons, calcium release from the ER is mediated either by IP3R or by RYR activation [10]. In insect DUM neurons, RYRs can be reliably blocked by intracellular application of 100 nM dantrolene [19]. To further prove the effectiveness of intracellular dantrolene in our experiments, we activated RYRs by bath application of caffeine, imaged the resulting calcium signal caused be RYR activation, and then demonstrated that dantrolene completely blocked calcium release in responses to caffeine (see Figure S1). However, dantrolene had no effect on voltage-induced increases in [Ca^2+^]_i_ under calcium-free conditions (Figure 3), demonstrating that this effect was not mediated by RYRs. Under calcium-free extracellular conditions, calcium inward current was zero, but an increase in intracellular calcium indicator fluorescence of 32 ± 6% amplitude still occurred in response to the depolarizing current step (Figure 3B).

**Figure 4:**
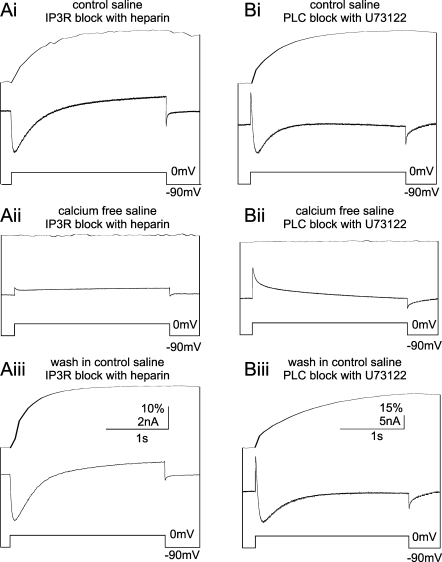
Voltage-Induced Intracellular Calcium Signals Depend on IP3R and PLC Activation (A) (i) In heparin-loaded (500 nM), acutely isolated DUM neurons in TEA- and TTX-containing saline, a voltage step from −90 mV to 0 mV causes a calcium inward and a calcium-activated potassium outward current accompanied by an intracellular calcium signal. (ii) Exposing the heparin-loaded cell for 3 min to zero-calcium saline causes a complete loss of all membrane currents and the calcium signal. (iii) Switching back to calcium-containing saline for 3 min restores the original currents and the calcium signal. (B) Same representative experiment as in (A), but the neuron is loaded with the PLC blocker U73122. Pharmacological block of IP3Rs and of PLC both abolish voltage-induced calcium signals in the absence of calcium inward current.

In contrast, intracellular application of the IP3R-blocker heparin completely abolished voltage-dependent calcium release from internal stores in the absence of extracellular calcium (Figure 4A). Heparin has been shown to block insect DUM neuron IP3Rs at micromolar concentrations [19]. We have further demonstrated the effectiveness of heparin in our experiments by demonstrating that it blocks intracellular calcium release induced by bath application of the PLC agonist m-3M3FBS (25 μM; Calbiochem, San Diego, California, United States; see Figure S2). Loading the neurons intracellularly with heparin (0.5 μM) via the patch pipette had no effect on calcium currents recorded in voltage clamp (Figure 4Ai). Under zero extracellular calcium conditions, heparin-loaded cells showed no increase in [Ca^2+^]_i_ as a response to membrane depolarizations. After 3 min in calcium-free saline, no voltage-activated calcium inward current and no increases in fluorescence were observed (Figure 4Aii). Washing for 3 min in calcium-containing saline completely restored the initial voltage-activated calcium current and the resulting increases in fluorescence (Figure 4Aiii). These data showed that membrane depolarization–induced intracellular calcium release without calcium influx depended on IP3R activation. These pharmacological data were further substantiated by the finding that voltage-induced intracellular calcium signals in the absence of calcium influx could be blocked by bath application of the membrane-permeable IP3R blocker 2-aminoethoxydiphenyl borate (2-APB). However, the effect of 2-APB was only partially reversible in locust DUM neurons (see Figure S3).

IP3 is produced by phospholipase C (PLC) activity. In insect DUM neurons, PLC activity can be specifically blocked by U73122 [20]. Intracellular application of U73122 (Figure 4B) led to the same results as IP3R block. In calcium-containing saline PLC block had no effect on calcium inward currents (Figure 4Bi). Under zero extracellular calcium conditions, U73122-loaded cells showed no intracellular calcium signal in response to membrane depolarizations (Figure 4Bii). Washing for 3 min in calcium-containing saline completely restored the initial voltage-activated calcium current and the resulting increases in fluorescence (Figure 4Biii). This demonstrated that voltage-induced calcium release from intracellular stores in the absence of calcium influx was mediated via the classical PLC/IP3R pathway [10].

### Voltage-Induced G-protein Activation Is Necessary for the Calcium Signal

In neurons, activation of many different G-protein–coupled receptors may activate PLC. In our experiments, activation of any of these receptors was impossible, because the neurons were freshly isolated, they were patched and imaged prior to neurite outgrowth, they had no contact with other neurons, no transmitters or signaling substances that could possibly activate any receptor located on the plasma membrane were added to the saline, and a constant flow of fresh saline was washed over the isolated somata throughout the experiments. Furthermore, calcium signals that occurred in the absence of calcium influx were time locked to imposed membrane depolarizations, and spontaneous depolarizations or baseline fluctuations of comparable amplitude never occur in freshly isolated DUM neurons. Due to the lack of synaptic specializations and the constant saline flow, it seemed unlikely that depolarizations to 0 mV might have caused release of the natural transmitter octopamine from freshly isolated DUM somata, which in turn, might have activated autoreceptors. Nevertheless, we repeated the experiments shown in Figure 1 under pharmacological block of octopamine receptors (epinastine [21]). This had no effect on the calcium responses, showing that activation of octopamine autoreceptors was not the cause for PLC/IP3R–mediated intracellular calcium release (unpublished data).

**Figure 5:**
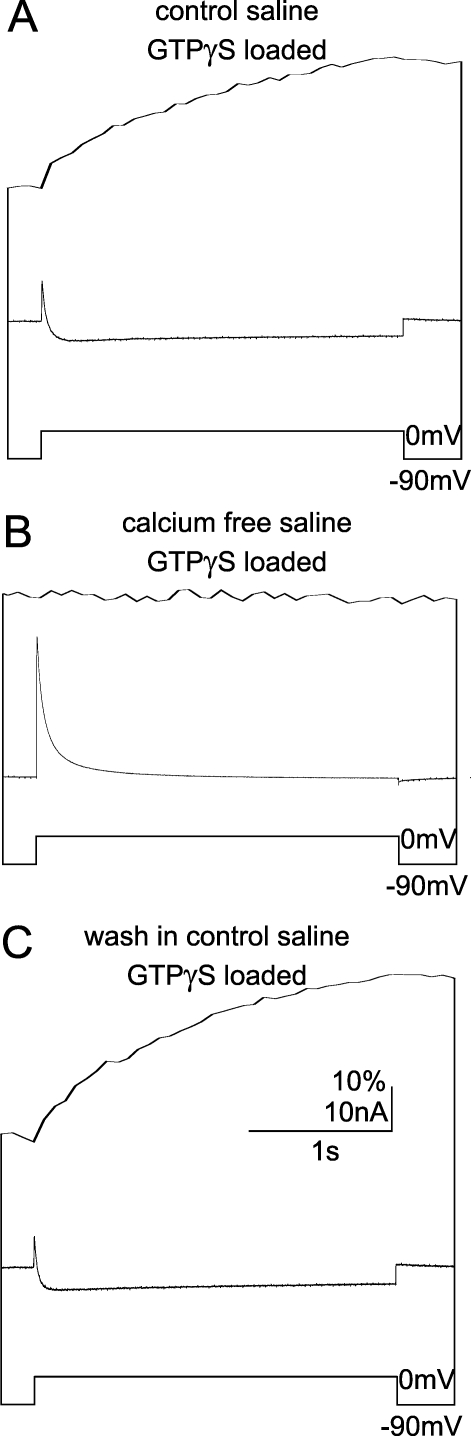
Voltage-Induced Intracellular Calcium Signals Depend on G-protein Activation Acutely isolated neurons are loaded with GTPγS (100 μM), and a voltage step from −90 mV to 0 mV is repeated 30 times in calcium-containing saline to minimize availability of native G-protein. (A) In calcium-containing saline depolarization causes calcium inward current accompanied by an intracellular calcium signal. (B) Exposing the GTPγS-loaded cell for 3 min to zero-calcium saline causes a complete loss of membrane currents and the calcium signal. (C) Switching back to calcium-containing saline for 3 min restores the original current and the calcium signal.

Therefore, PLC was activated by the depolarization of the plasma membrane, and not through ligand activation of a G-protein–coupled receptor. To test whether PLC was activated by a G-protein, the neurons were loaded with a non-hydrolysable GTP analog (GTPγS, 100 μM). Consequently, activated G-proteins coupled to GTPγS retained separated subunits and could not be made available for future activations. To saturate native intracellular G-proteins with GTPγS, neurons were loaded with GTPγS via the patch pipette (100 μM) and depolarized 30 times for 2.5 s each in calcium-containing saline (Figure 5A). In calcium-containing saline, the neurons could be depolarized several hundred times without depleting intracellular calcium stores. Switching to zero-calcium saline completely abolished the calcium inward current as described above. However, in GTPγS-loaded neurons, no voltage-induced increase in calcium indicator fluorescence was observed in the absence of calcium inward current (Figure 5B). Washing in calcium-containing saline restored both the calcium current and also the resulting calcium signals (Figure 5C). The saturation of native intracellular G-protein could also be fully accomplished by 25 depolarizations of GTPγS-loaded cells in calcium-containing saline, so that no calcium signal was observed in subsequent test depolarizations in zero-calcium saline. Using only 20 depolarizations of GTPγS-loaded neurons in calcium-containing saline prior to the test depolarization in calcium-free saline yielded approximately 75% reduction in the signal amplitude of voltage-induced calcium release in the absence of calcium inward current (unpublished data). In summary, the data showed that step membrane depolarizations from −90 to 0 mV induced calcium release from internal stores which occurred in the absence of calcium influx and relied on G-protein (Figure 5), PLC, and IP3R (Figure 4) activation. Moreover, calcium elevations without any calcium influx also occurred during spike trains as observed during normal behavior (Figure 2).

### Voltage Dependency of Novel Calcium Signaling Mechanism

**Figure 6:**
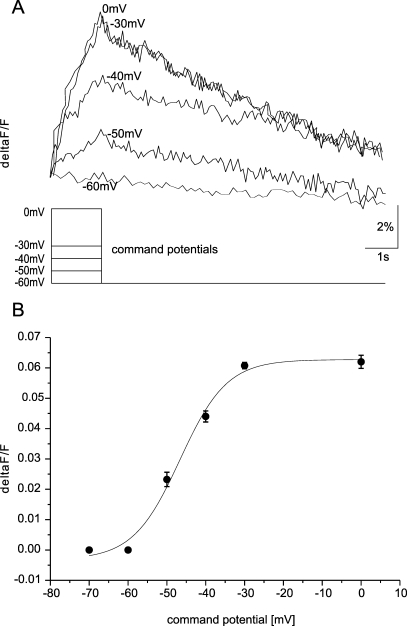
Voltage Dependence of the Calcium Signal (A) Acutely isolated DUM neurons are maintained in calcium-free saline containing TTX and TEA, and clamped to a holding potential of −60 mV followed by command potentials of −50, −40, −30, and 0 mV. The amplitudes of the resulting intracellular calcium signals change as a function of the command potential. (B) Percent change (means and standard deviations from three neurons) in intracellular calcium levels (*y*-axis) plotted as a function of command voltage (*x*-axis).

In a final set of experiments, we determined the voltage dependency of this novel calcium-release pathway. In order to avoid depletion of internal calcium stores during the course of the experiments, in a first set of measurements, the activation voltage was roughly narrowed down, and in a second set of measurements, the voltage dependency was determined more accurately within the pre-determined voltage range. First, in calcium-free saline, neurons were clamped to −90 mV followed by test steps to −60 mV, −30mV, and 0 mV. Calcium signals were not caused by depolarizations to −60 mV, but by those to −30 mV and to 0 mV (unpublished data). Second, in calcium-free saline neurons were clamped to −60 mV, followed by test pulses to −50, −40, −30, and 0 mV (Figure 6A), and the percentage increase in fluorescence was averaged over three neurons and plotted as a function of the command voltage (Figure 6B). The threshold for G-protein–PLC-IP3–mediated calcium release without calcium influx was between −60 mV and −50 mV. This was followed by a nearly linear increase of the calcium signal amplitude for command potentials between −50 mV and 0 mV. Command potentials more positive than −30 mV did not further increase the calcium signal amplitude (Figure 6B).

## Discussion

Our data clearly demonstrate G-protein activation upon plasma membrane depolarization, which in turn activates PLC and IP3R to cause calcium release from the ER. The mechanism by which membrane voltage is translated into G-protein activation remains to be unraveled. One possibility to consider might be intracellular signaling by ions entering the neurons via voltage-operated channels. In addition to calcium [10], chloride [22] and sodium [23] also act as intracellular messengers. They seem unlikely, however, as no membrane currents were recorded in calcium-free saline also containing pharmacological agents to block voltage-operated sodium and potassium currents. Furthermore, influx of small amounts of “rest calcium” in the absence of calcium current can be excluded, because the calcium-activated potassium current also disappeared, and additional blockade of VOCs by cadmium did not affect the intracellular calcium signal. Chloride influx is unlikely because voltage-dependent chloride channels activating at depolarizing current steps are not present in DUM neurons [15,24]. Sodium channels were blocked with TTX, but in the absence of calcium, sodium may also pass VOCs [25]. Sodium-induced dissociation of G-protein subunits has been demonstrated in neurons [26]. However, neither pharmacological block of VOCs by cadmium or by magnesium, nor activation of voltage-dependent sodium channels (leaving out TTX) affected the amplitude of the intracellular calcium signal. Therefore, we conclude that G-protein activation was not mediated by ion influx that was hidden to our patch clamp recordings.

Alternatively, G-protein activation might be mediated by interactions with the voltage-dependent subunit of a membrane protein complex. During recent years, it has become evident that most ion channel pore-forming subunits interact with several modulatory binding partners to form a dynamic signaling protein complex [27]. For example, the G-protein β-γ heterodimer binds directly to VOCs (and also to potassium channels), and the channel sites have been mapped in detail [28]. In *Caenorhabditis elegans,* the potassium channel β-subunit, MPS-1, is also a serine/threonine kinase [29], and multiple regulatory proteins bind to *Drosophila* BK channels [30,31]. Research in this field has been channel-centric, assuming that these interactions exist only to regulate the properties of the membrane channels, but the possibility of information flow from the channel protein to the associated protein complex has also to be considered [27]. This is further supported by findings in non-excitable megakaryocytes, in which IP3R-dependent calcium release induced by purinergic receptors (P2Y1) can be potentiated by membrane depolarizations [32]. Additional evidence for voltage-dependent G-protein activation comes from cultured arterial myocytes [33], and a recent study on transfected fibroblasts suggests MAP kinase activation depending on the conformational state of the voltage sensor of ether-a-go-go potassium channels [34]. The current study demonstrates the existence of voltage-dependent G-protein activation for the first time in neurons. Since neurons process information by membrane voltage changes, the consequences of such a signaling pathway for brain development and function may be multi-fold and hard to foresee at this point. The voltage dependency of the intracellular calcium signal observed in this study follows a Boltzmann kinetic that closely resembles the activation characteristics of VOCs in insect DUM neurons [15]. Although we have no further evidence for direct interactions between the VOCs and a G-protein, G-protein activation via a highly sensitive voltage-sensing protein located in the plasma membrane remains the most likely explanation for our data.

What are the functions of voltage-dependent calcium release from the ER that does not rely on calcium influx? Neurons possess a rich blend of different types of VOCs located in different cellular compartments [10]. This ensures voltage-dependent calcium influx at highly specialized locations to regulate vital processes such as vesicle fusion at synaptic terminals [7], filopodia morphology and motility [5], or local spine stability at postsynaptic sites. However, neurons most certainly also possess dendritic or axonal sites that are devoid of VOCs, but become depolarized either passively or actively by sodium influx during normal neural function. The novel mechanism described here might enable neurons to locally monitor membrane depolarizations by intracellular calcium release at such sites. The ER, in turn, is organized in modular signaling units capable of performing independent functions ranging from the local activation of kinases, phosphatases, or SOCs (via local protein–protein interactions of the ER and the plasma membrane) to the activation of stress signaling pathways and transcription [35]. The fundamental new aspect resulting from this study is that the ER can acknowledge neuronal activity by local calcium release without relying on calcium as an input signal. Such a voltage-dependent intracellular calcium signal is not a unique peculiarity of locust DUM neurons, but strong evidence for its existence has recently also been observed in other neurons of the invertebrate CNS where it triggers BK channel activation (P. Kloppenburg, personal communication).

Conversely, instead of a spatial separation of different intracellular calcium signals, calcium influx through VOCs or through ROCs may act in concert with voltage-induced activation of PLC. In hippocampal neurons, for instance, IP3R activation is evoked by synaptic activation of metabotropic glutamate receptors paired with back-propagating action potentials [13]. The IP3R is well suited to operate as a coincidence detector, because its sensitivity for calcium is altered by IP3. In general, IP3Rs show a bell-shaped calcium dependence, but in the presence of high IP3, their calcium dependence becomes sigmoidal [36,37]. Consequently, a calcium-independent voltage sensor activating PLC to produce IP3 should tune IP3Rs towards responding to higher calcium concentrations. Although the physiological function of this novel calcium-release mechanism for normal neural function remains to be investigated, it clearly expands the calcium signaling tool kit of neurons to facilitate either calcium release from the ER without calcium as input signal or coincidence detection of multiple activity-dependent signals on the level of the IP3 receptor.

## Materials and Methods

### Cell isolation.

Adult desert locusts Schistocerca gregaria were obtained from the laboratory culture of the Department of Neurobiology at the Free University of Berlin. Animals were dissected and DUM neurons were isolated as described earlier [15]. Only acutely isolated cells were used for measurements (neurons older than 4 h were discarded). To exclude cell–cell interactions, all neurons recorded from had no contact with other neurons or glia cells, nor was any other cell located within a 100-μm circumference (see Figure 1G–1I). Locust thoracic DUM neurons can be subdivided into different subclasses [15,18]. All types of thoracic DUM neurons show a slow, non-inactivating calcium current and a fast, inactivating calcium current, but different subtypes may show different relative amounts of both calcium currents. However, the novel calcium signaling mechanism described in this manuscript is present in all subclasses of locust thoracic DUM neurons.

### Immunocytochemistry and microscopy.

Acutely isolated neurons were fixed in 4% paraformaldehyde, washed in PBS buffer (0.1 M; 3 × 10 min), incubated for 15 min at 37 °C in RNase (0.1 mg/ml buffer) to diminish RNA, and washed 3 × 10 min in PBS buffer. This was followed by blocking with NGS (10% in PBS) for 60 min. Then anti-HRP antibody (AffiniPure Rabbit Anti-Horseradish Peroxidase; Dianova, Hamburg, Germany) was applied in PBS (1:5.000) overnight. This was followed by six washes in PBS (10 min each), 20-min incubation with propidium iodide (50 μl of a 500 μM stock solution/l PBS), six washes in PBS (5 min each), 60-min incubation of Cy2-goat anti rabbit secondary antibody (1:1,000 in 0.1 M PBS, Dianova), an additional six washes in PBS (5 min each), and mounting in glycerol. Confocal images were obtained with a 40× oil immersion lens on a Leica SP2 confocal microscope (Leica, Wetzlar, Germany). Cy2 was excited with an argon laser at 488 nm, and the emission was detected between 495 and 520 nm. PI was excited with a green helium neon laser at 543 nm, and emission was detected between 560 and 590 nm.

### Solutions.

The standard external saline contained (in mM): 150 NaCl, 5 KCl, 5 CaCl_2_, 2 MgCl_2_, 10 Hepes, 25 sucrose, adjusted to pH 7.40 with NaOH. In all voltage clamp experiments, 300 nM tetrodotoxin (TTX; Sigma, St. Louis, Missouri, United States) was added to block Na^+^ currents. To reduce K^+^ current amplitudes at the test potential of 0 mV, high-K^+^ saline and tetraethylammonium chloride (TEA-Cl) were used for most experiments (in mM): 50 KCl, 35 NaCl, 5 CaCl_2_, 2 MgCl_2_, 70 TEA-Cl, 10 Hepes, 25 sucrose. For Ca^2+^-free saline, CaCl_2_ was replaced by an equal concentration of MgCl_2_ and 1 μM EGTA was added. Calcium-free extracellular solution was achieved by switching the saline flow from calcium-containing to calcium-free saline. At a flow rate of 1 ml per minute, the bath volume of approximately 500 μl was exchanged ten times within 5 min. The standard pipette solution contained (in mM): 180 K gluconate, 10 NaCl, 0.1 CaCl_2_, 1 MgCl_2_, 10 Hepes, 2 ATP-Mg, 1 EGTA. The pH was adjusted to 7.40 with KOH.

### Cell staining and electrical recording.

Dye filling and recording were conducted with different electrodes to exclude changes in intracellular dye concentration during the course of the experiments. For cell staining, the tips of thick-walled micropipettes (resistance, 20–25 MΩ) were filled with 350 μM Oregon Green Bapta-2 octapotassium salt (Invitrogen, Carlsbad, California, United States) in standard pipette solution. The shafts were filled with 2 M potassium acetate with an air bubble left between the dye and the potassium acetate. Neurons were dye loaded iontophoretically by applying hyperpolarizing current of 1–1.5 nA amplitude for 5 min. After dye loading, the electrode was removed and the cells were patched.

Whole-cell patch clamp recordings were carried out using an EPC-9 patch clamp amplifier (HEKA Elektronik, Lambrecht, Germany) in the voltage-clamp and current-clamp mode. PULSE 8.30 software (HEKA Elektronik) was used to generate voltage steps or to inject current. Patch pipettes were pulled from filamented borosilicate glass capillaries (Harvard Apparatus, Edenbridge, United Kingdom) with an outer diameter of 1.5 mm (resistance, 1–2 MΩ). Series resistance compensation of 75%–80% was achieved. Liquid junction potential was corrected before conducting a giga seal. Recordings were analyzed using PULSEfit 8.30 software and Igor pro 5.2 software. Leak correction was conduced off-line.

### Imaging.

Oregon Green Bapta-2 octapotassium salt was excited at 480-nm wavelength. The fluorescent images were captured through a 510- to 530-nm band-pass filter with a cooled CCD camera (Hamamatsu 4742–95; Hamamatsu Photonics, Hamamatsu City, Japan) mounted on a fluorescence microscope (Zeiss Axioskop 2FS; Carl Zeiss, Oberkochen, Germany). Data acquisition and analysis were conducted with Simple PCI software (Compix, Sewickley, Pennsylvania, United States). Excel 4.0 (Microsoft, Redmond, Washington, United States) and Microcal Origin 7.0 (Microcal Software, Northampton, Massachusetts, United States) were used for statistical analysis.

### Data analysis.

Currents that resulted from voltage steps from −90 mV to 0 mV are shown together with the underlying K^+^ currents, Na^+^ currents were potently blocked by application of 300 nM TTX. Ca^2+^ currents resulting from the performed voltage steps were isolated by an off-line subtraction protocol using PULSEfit software (HEKA Elektronik). Currents were leak corrected by off-line subtraction routines. Calcium signals were always depicted as percentage change in the fluorescence (ΔF/F). Background fluorescence was subtracted routinely. Background fluorescence was measured as the average fluorescence of three randomly chosen regions of interest located between 50 and 70 μm outside the DUM neuron soma of interest. Average background fluorescence was subtracted in each frame of every time series from the fluorescence signal of the calcium indicator Oregon Green Bapta measured inside the DUM neuron somata. Baseline correction for bleaching was not necessary.

## Supporting Information

Figure S1 Dantrolene Blocks Calcium Signals Evoked by Bath Application of Caffeine Freshly isolated neurons were filled with Oregon Green Bapta via a sharp microelectrode. After successful dye loading, the electrode was removed to avoid further loading during the experiments. Bath application of the RYR agonist caffeine (20 mM) induced a large increase in the fluorescence of the calcium indicator (light gray trace). Ten minutes after patching the same cell with dantrolene (100 nM) in the pipette solution, the calcium response to bath application of caffeine was markedly reduced (dark gray trace). Twenty minutes after intracellular perfusion with dantrolene via the patch pipette, that response to caffeine was completely blocked (black trace). The same results were obtained in five experiments. Control experiments with no dantrolene in the patch pipette demonstrated that large calcium signals were always observed in response to caffeine bath application 10, 20, 30 and 40 min after patching onto the neurons.
(5.8 MB TIF)

Figure S2 Heparin Blocks IP3-Mediated Calcium Release Induced by Pharmacological Activation of PLC Freshly isolated neurons were filled with Oregon Green via a sharp microelectrode. After successful dye loading, the electrode was removed to avoid further loading during the experiments. The neurons were patched with heparin in the patch pipette (0.5 μM), and bath application of the PLC agonist m-3M3FBS (Calbiochem) was conducted within the first minute after the seal, before the neurons were loaded with heparin. Bath application of m-3M3FBS (25 μM) induced a 20% increase in the fluorescence of the calcium indicator (gray trace). After 10 min, the neurons were loaded with the IP3R blocker heparin, and bath application of m-3M3FBS did not induce any calcium signal (black trace).
(4.6 MB TIF)

Figure S3 Voltage-Induced Intracellular Calcium Signals in the Absence of Calcium Influx Can Be Blocked by 2-APB Cells were filled with calcium indicator (Oregon Green Bapta) and clamped at −90 mV holding potential. In calcium-containing saline, a large increase in the fluorescence of the calcium indicator was observed (light gray trace) in response to a voltage step to 0 mV for 2.5 s (see inset). After 5 min in calcium-free saline, the voltage step induced a smaller calcium signal (trace 1). This signal was completely blocked after 5 min of bath application of 2-APB (trace 2). The signal could only be partially restored after 5 min of washing with calcium-free saline (trace 3).
(1.5 MB TIF)

## Abbreviations

[Ca^2^^+^]i: free intracellular calcium
DUM: dorsal unpaired median
ER: endoplasmic reticulum
IP3R: inositol 1,4,5-trisphosphate receptor
PLC: phospholipase C
RYR: ryanodine receptor
VOC: voltage-operated calcium channel
